# Peculiar Evolutionary History of miR390-Guided TAS3-Like Genes in Land Plants

**DOI:** 10.1155/2013/924153

**Published:** 2013-11-02

**Authors:** Maria S. Krasnikova, Denis V. Goryunov, Alexey V. Troitsky, Andrey G. Solovyev, Lydmila V. Ozerova, Sergey Y. Morozov

**Affiliations:** ^1^Belozersky Institute of Physico-Chemical Biology, Lomonosov Moscow State University, Moscow 119991, Russia; ^2^Main Botanic Garden Russian Academy of Sciences, Botanicheskaya 4, Moscow 127276, Russia

## Abstract

PCR-based approach was used as a phylogenetic profiling tool to probe genomic DNA samples from representatives of evolutionary distant moss taxa, namely, classes Bryopsida, Tetraphidopsida, Polytrichopsida, Andreaeopsida, and Sphagnopsida. We found relatives of all *Physcomitrella patens* miR390 and TAS3-like loci in these plant taxa excluding Sphagnopsida. Importantly, cloning and sequencing of *Marchantia polymorpha* genomic DNA showed miR390 and TAS3-like sequences which were also found among genomic reads of *M. polymorpha* at NCBI database. Our data suggest that the ancient plant miR390-dependent TAS molecular machinery firstly evolved to target AP2-like mRNAs in Marchantiophyta and only then both ARF- and AP2-specific mRNAs in mosses. The presented analysis shows that moss TAS3 families may undergone losses of tasiAP2 sites during evolution toward ferns and seed plants. These data confirm that miR390-guided genes coding for ARF- and AP2-specific ta-siRNAs have been gradually changed during land plant evolution.

## 1. Introduction

**Figure 1:**
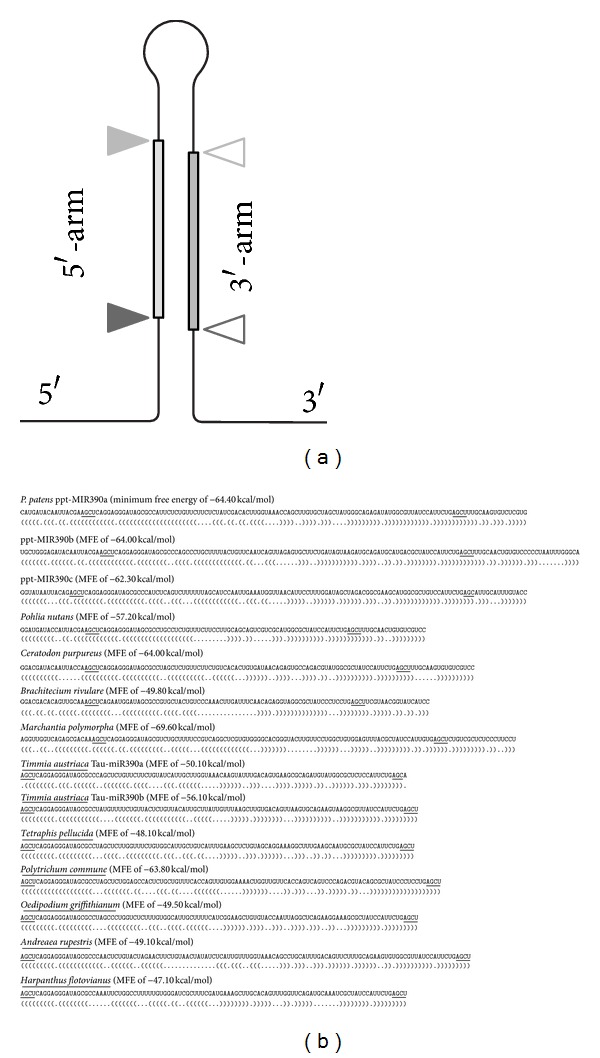
(a) Schematic representation of a typical miRNA hairpin showing the position of the lower DCL processing site (filled and open dark gray arrowheads), upper DCL processing site (filled and open gray arrowheads), and the positions of potential mature miRNA or miRNA* (filled and open arrowheads, resp.). (b) Primary and predicted secondary structures of stem-loop elements in Bryophyta and Marchantiophyta miR390 precursors. Sequences of the highly conserved AGCU tetranucleotides at the lower DCL processing sites of miRNA or miRNA* are underlined. Names of plant species with sequenced portions limited by conserved AGCU tetranucleotides are underlined.

MicroRNAs (miRNAs) are a class of small interfering RNAs (siRNAs) that are 21–24 nt in length and predominantly function to repress gene expression at posttranscriptional level. Many miRNAs found to be ubiquitously present in a wide range of animals, plants (including algae), and some animal viruses [1–7]. Various miRNA families were found to be highly conserved across the plant kingdom [3, 7–10]. Plant miRNAs are transcribed by RNA polymerase II to generate primary capped and polyadenylated miRNA (pri-miR) transcripts. These pri-miRs range from few hundred bases to several thousand bases and contain a partly complementary double-stranded hairpin structure [1, 3, 4, 7, 8, 11, 12]. A nonperfect duplex of 21 nt RNAs (miRNA/miRNA* duplex), with characteristic two-nucleotide 3′ overhangs is excised from this hairpin precursor by a DCL (Dicer-like) protein and accessory factors (Figure 1(a)). Each strand of duplex is methylated on its 3′ nucleotide by HEN1. Then one strand of the excised duplex is incorporated into a complex containing an AGO slicer protein, thereby becoming a mature miRNA [1, 3, 8, 9, 11, 12]. 

Highly conserved plant miRNA, miR390, forms a specialized complex with slicer AGO7 [13]. The activity of the latter protein is required to stably associate this complex with the 5′ site and cleave the 3′ site of miR390 dual target sites in TAS3 precursor transcripts to start pathway resulting in trans-acting siRNAs (ta-siRNAs) [1, 14, 15]. Following miRNA-directed cleavage, tasiRNA precursor transcripts enter into an RDR6- and SGS3-dependent pathway and are processed by DICER-like4 (DCL4) protein into phased, 21 nucleotide ta-siRNAs [16]. In general, ta-siRNAs are subclass of so-called phasiRNAs [1, 14, 15]. In dicots, the TAS3 ta-siARF RNAs regulate leaf patterning, developmental timing, and rate of lateral root growth by repressing the Auxin Response Factors (ARF-2, -3 and -4) [17–19]. It is currently known that many important processes of plant development and physiology (including those in mosses, see [20, 21]) are regulated by numerous classes of phasiRNAs [1, 14, 15]. The discovery that organisms share similar “developmental genes” as well as fundamental aspects of their ontogeny opens the door towards a molecular understanding of development using comparison of gene structures. Thus, orthologous genes may be studied in species occupying key phylogenetic positions to deduce correlations between the molecular changes that were responsible for evolutionary events in distinct species of interest [22].

Previously, we described a new method for identification of plant ta-siARF precursor genes based on PCR with oligodeoxyribonucleotide primers mimicking miR390. The method was found to be efficient for genomic DNA and cDNA of dicotyledonous plants, cycads, and conifers [23]. The PCR-based approach was used as a comparative profiling tool to probe genomic DNA samples from species belonging to classes Bryopsida and Sphagnopsida, although it is only partly suitable for a comprehensive description of all TAS3 genes in a particular organism [24]. Previously, there were no other papers connected to phylogenetics aspects of TAS3 genes in species outside flowering plants, namely, pines, cycads, ferns, horsetails, clubmosses, and liverworts. In mosses, the only plant, where TAS genes were studied in details, was *Physcomitrella patens* (see below). This paper combines our current data and new findings of other research groups to uncover a peculiar picture of an evolution of TAS3-like genes. 

## 2. Materials and Methods

### 2.1. Plant Material

Plant material was taken from the collections of the N.V. Tsytsin Main Botanical Garden of the Russian Academy of Sciences and the Biological Faculty of M. V. Lomonosov Moscow State University. 

### 2.2. Analysis of Nucleic Acids

Genomic plant DNA was isolated from 200 mg of fresh or dried plant material by DNA extraction kit (Macherey-Nagel) according to the protocol of the manufacturer. TAS3 genes were amplified and sequenced as described in [23, 24]. DNA sequences were deposited at the NCBI data bank, and the accession numbers are shown in Table 1.

Total RNA was isolated from green parts of plants with the Trizol reagent according to the manufacturer's instructions (Invitrogen). Digestion of any contaminating DNA was achieved by treatment of samples with RQI RNase-free DNase (Promega). Reverse transcription was performed with 1 **μ**g of total RNA and oligo (dT)-primer t20-xho (attctcgaggccgaggcggccgacatgtttttttttttttttttttttttttv) using the RT system (Invitrogen) according to the protocol of the manufacturer. Primers for dicotyledonous plants were forward primer TAS-P (5′-GGTGCTATCCTATCTGAGCTT-3′) and mixture of reverse primers TAS-Mcaa (5′-AGCTCAGGAGGGATAGCAA-3′) and TAS-Maca (5′-AGCTCAGGAGGGATAGACA-3′). For PCR, 25–35 cycles were used for amplification with a melting temperature of 95°C, an annealing temperature of 58°C, and an extending temperature of 72°C, each for 30 seconds, followed by a final extension at 72°C for 3 min. PCR products were separated by electrophoresis of samples in 1.5% agarose gel and purified using the GFXTM PCR DNA and Gel Band Purification Kit (Amersham Biosciences). For cloning, the PCR-amplified DNA bands isolated from gel were ligated into pGEM-T (Promega). Cloned products were used as templates in sequencing reactions with the ABI Prism BigDye Terminator Cycle Sequencing Ready Reaction Kit (Applied Biosystems). DNA and cDNA sequences were deposited at the NCBI data bank, the accession numbers are JN692262, JN692261, JN692260, and JN692259. 

### 2.3. Computational Sequence Analysis

TAS3-like sequences identified in this paper and found in the NCBI database were compared using multiple alignment tool at MAFFT (version 5.8). The phylogenetic tree was generated according MAFFT6 program (http://mafft.cbrc.jp/alignment/server/). Sequences were additionally analysed at http://blast.ncbi.nlm.nih.gov/BlastAlign.cgi. 

## 3. Results and Discussion

### 3.1. Diversity of miR390 and TAS3-Like Genes in Land Plants

Sequence data for TAS3 genes of dicotyledonous [23, 27] and monocotyledonous plants [28] have revealed two TAS3 variants, two- and one-tasiARF subfamilies, respectively. The first subfamily displays canonical structural features well characterized for AtTAS3 in *Arabidopsis thaliana* [29]; the tandem of conserved ta-siARF sequences are flanked by a constant miR390-targeted site at the 3′ end and a divergent (noncleavable) region at the 5′ end. In contrast, transcripts of the second subfamily encode only single copy of tasiARF. Importantly, there was no mismatch in the tenth position of the 5′ miR390 target site, suggesting that the 5′ miR390 binding site may undergo cleavage for initiation of TAS3 precursor RNA processing [14, 15, 27]. Moreover, these shorter TAS3 genes, despite the fact that they share the presence of the dual miR390 with two-tasiARF subfamily, have the lesser conserved regions flanking the tasiARF area in reverse orientation, with a constant region at the 5′ end and a divergent region at the 3′ end [23, 27]. 

It is well known that the species of miR390 function as activators of a ta-siARF pathway [1, 3, 14, 15]. In plants, most miRNA-encoding loci comprise independent, nonprotein-coding transcription units. miRNA genes are transcribed by RNA polymerase II (pol II). The primary miRNA transcripts (pri-miRNAs) contain cap structures as well as poly(A) tails. Like protein-coding genes, promoters of miRNA loci contain canonical cis-promoter elements, such as TATA box and transcription initiator, and various transcription factor responsive elements [30]. Plant pre-miRNA hairpins sometimes occur in genomic clusters, strongly suggesting expression of multiple hairpins from a single pri-miRNA. Most clusters in these species (61% to 90%) contain hairpins encoding identical mature miRNAs, suggesting that they were the result of local tandem duplications and serve to increase the dosage of a particular miRNA from a single promoter [1, 9, 31]. 

Gymnosperms and angiosperms are believed to diverge from a common ancestor >325 million years ago [32]. Many miRNAs are common between the two phyla [3, 8, 33]. This correlates with an obvious conservation of the TAS3 and miR390 loci between these plant groups [6, 9, 15]. On the other hand, evolutionary history of TAS3-like and miR390 genes in more anciently diverged pteridophytes remains enigmatic. 

Bryophytes *sensu lato* (liverworts, mosses, and hornworts) are placed as a phylogenetic grade between the charophycean algae and pteridophytes [25]. It is believed that the ancestors of bryophytes and vascular plants separated shortly after the transition from water to land [34]. Bryophytes like moss *Physcomitrella patens* are thought to closely resemble the first land plants in their gametophyte-dominated life cycles, lack of lignified vascular tissue, and morphology. Some abundant miRNAs are invariant between basal plant Physcomitrella and more recently derived lineages. Particularly, at least 11 miRNA families, including miR390, are conserved between *P. patens*, *A. thaliana, *and *O. sativa *[31, 35, 36]. In the case of miR390, three genomic loci were revealed in *P. patens* [37]. Recently, this moss has been shown to code for six precursor ta-siARF loci targeted by miR390 and referred to as PpTAS3a–f [15, 20, 36, 38]. All six loci contain two (5′ and 3′) miR390-target sites and monomeric ta-siRNA sequences, which regulate ARF genes and also target mRNA of AP2-related transcription factors [20].

#### 3.1.1. Search for Novel miR390 Genes in Bryophyta and Marchantiophyta

Assuming that there is the only nonvascular plant (*P. patens*) with miR390 genes identified and sequenced [31], we performed their search in liverworts and other mosses. Identification of miRNA largely relies on two main strategies: computer-based (bioinformatics) and experimental approaches. Search for miRNA genes using bioinformatics tools is one of the most widely used methods, contributing considerably to the prediction of new miRNAs in different systems. The main theory behind this approach is finding signature sequences and secondary structures of known miRNAs both within a single genome and across genomes of related organisms [39]. It is well known that miRNAs are well conserved from species to species within the same plant taxa, which allows researchers the ability to predict orthologues of previously known miRNAs by utilizing EST and SRA NCBI databases (http://www.ncbi.nlm.nih.gov/). Interestingly, it was recently found that after the appearance of the miR390 family the gene duplication, divergence, and neofunctionalization gave rise to at least seven families of new miRNAs in two major superfamilies [15]. 

Availability of two moss cDNA databases (*Pohlia nutans* and *Ceratodon purpureus*) in addition to *P. patens* suggested identifying new Bryopsida miRNA genes using Physcomitrella as a reference. We used a method principally similar to that described by Jones-Rhoades and Bartel [39]. The first step for identifying miR390 precursors in the moss transcriptomes was detecting sequence reads containing imperfect inverted repeats corresponding to sequences of miR390 and miR390* using the “blastn.” Importantly, we used all combination of somewhat divergent miR390/miR390* sequences found for *P. patens* (http://www.mirbase.org/) (Figure 1). Second, the RNA-fold (http://rna.tbi.univie.ac.at/cgi-bin/RNAfold.cgi) program was used to find potential miRNA hairpin structures. Then strict criteria were adopted in the identification of pre-miR390-like sequences from hairpin structure sequences [40]; namely, (1) 60 nucleotides is the minimum length of pre-miRNA sequences, (2) the stem of the hairpin structure (including the GU wobble pairs) includes at least 22 base pairs, (3) −35 kcal/mol should be the maximum free energy of the secondary structure, and (4) the secondary structure must not include multibranch loops. Both *Pohlia nutans* and *Ceratodon purpureus *databases showed single copies of the putative miR390 precursor genes capable of forming the stem-loop structures with a minimum free energy of −57.20 kcal/mol (NCBI accession number GACA01009225) and approximately 64 kcal/mol (NCBI accession number SRR074894.910234), respectively (Figure 1). The latter putative miR390 sequence had obvious similarity to miR390b of *P. patens* as it was revealed using software at http://www.mirbase.org/ (Figure 1 and data not shown).

**Figure 2:**
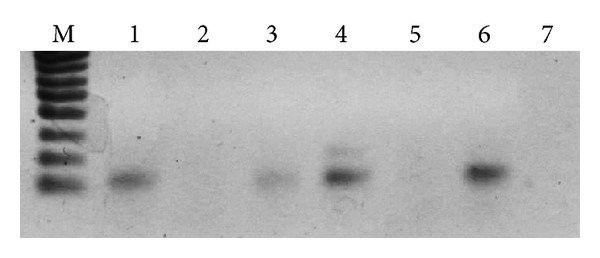
Analysis of PCR products in 1.5% agarose gel. Amplification of genomic DNA sequences flanked by miR390 and miR390* sites. PCR products were obtained on genomic DNAs with moss-specific primers [23, 24]. *Brachythecium rivulare *(1), *Nicotiana tabacum* (control) (2), *Marchantia polymorpha* (3), *Sphagnum squarrosum *(4), *Selaginella kraussiana *(control) (5), *Polytrichum commune* (6), and primers with no template DNA (control) (7). (M), DNA size markers including bands ranging from 100 bp to 1000 bp with 100 bp step and 1500 bp band (Sibenzyme).

 Computational methods for identifying miRNAs in plants are rapid and less expensive. However, these bioinformatic approaches can only identify conserved miRNAs among organisms where DNA or RNA sequence information is available. Efficient and suitable miRNA detection are essential to reveal miRNA precursors in organisms where sequence information is poor. We attempted to reveal miR390 sequences using our PCR-based approach [23] previously developed for TAS3 genes. PCR analysis was performed with a pair of degenerate primers; P-mir corresponded to the miR390 sequence and M-mir complementary to the miR390* region of Physcomitrella pre-miR390 hairpin-loop structure [8]. First experiment represented PCR reaction using *Brachythecium rivulare *(Bryopsida) DNA as template and degenerate primers. This reaction resulted in efficient synthesis of a single PCR-fragment with the expected size of 100 bp (Figure 2) that was in agreement with calculated distance between miR390 and miR390* sites in *P. patens *miR390 precursor RNAs (http://www.mirbase.org/). Sequencing of this cloned DNA fragment showed amplification of at least three genome loci (data not shown). Application of strict criteria that were used in the computer-based identification of pre-miR390-like sequences (see above) allowed us to select only single sequence capable of forming typical miRNA-like stem-loop structure (Figure 1). The introduction of next generation sequencing technology showed a powerful way for a more comprehensive exploration of miRNA gene repertoire. To confirm assignment of the revealed sequence to miR390, we performed blast search against *B. rivulare *Illumina genome sequence reads (to be published elsewhere). One of the reads showed 98% sequence similarity to the sequenced PCR fragment. Moreover, its foldback terminates at 17 bp below the miRNA/miRNA* region (Figure 1) that is typical for plant miRNAs, and this characteristic appears important for their optimal processing [8]. 

Further experiments were performed with moss species very distantly related to *P. patens*, namely, *Timmia austriaca* (class Bryopsida, subclass Timmiidae), *Tetraphis pellucida *(class Tetraphidopsida), *Bartramiopsis lescurii* (class Polytrichopsida), *Polytrichum commune* (class Polytrichopsida), *Andreaea rupistris* (class Andreaeopsida), *Oedipodium griffithianum* (class Oedipodiopsida), and *Sphagnum squarrosum* (class Sphagnopsida). PCR amplification of chromosomal DNA from all these species resulted in synthesis of one major fragment of ca. 100 bp (Figure 2 and data not shown). However, *S. squarrosum* showed additional minor fragment of 250 bp (Figure 2). Cloning and sequencing of the obtained DNA bands revealed that the one-third of amplified sequences contained putative miR390 sequences composed of stem-loop structure starting from miR390/miR390* regions corresponding to PCR primers. Moreover, some amplified sequences showed obvious similarity to miR390 from *P. patens* (data not shown). Interestingly, no one from 9 clones of *S. squarrosum *PCR products of 80–270 bp in length fit strict criteria for identification of pre-miR390-like sequences (see above). It is in accordance with our previous fail to find TAS3-like sequences in this plant [24]. 

**Figure 3:**
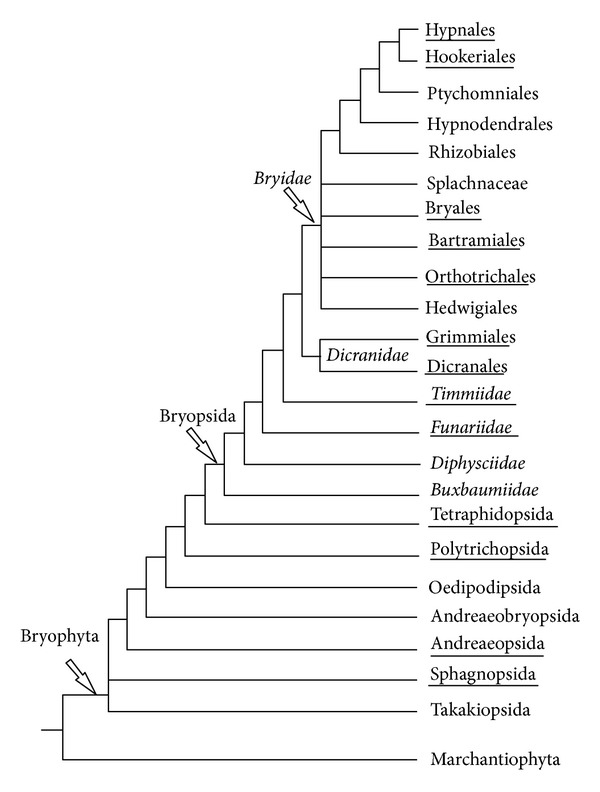
Synoptic consensus view on phylogeny of major Bryophyta and Marchantiophyta taxa based on recent molecular analyses, adopted from Figure 3 of Shaw et al. [25] with modifications. Taxa discussed in the current study are underlined.

 Assuming our promising results with mosses (see above) which are evolutionary distant from *P. patens*, we attempted to reveal miR390 sequences in liverworts *Marchantia polymorpha* (class Marchantiopsida) and *Harpanthus flotovianus* (class Jungermanniopsida) where these genes were not reported. PCR-based approach (see above) revealed miR390-like locuses in both species (Figures 1 and 2). These sequences were validated as miR390 precursor genes by MaturePred web server (http://nclab.hit.edu.cn/maturepred/) and secondary structure prediction at RNAfold web server (http://rna.tbi.univie.ac.at/cgi-bin/RNAfold.cgi). These potential liverwort miR390 genes fit strict criteria adopted for the prediction of pre-miRNA-like species from hairpin structures (see above). Using BLAST, we also revealed several sequence reads containing the PCR-tagged potential miR390 sequence in NCBI SRA database of *Marchantia* genome (see, i.e., NCBI accession number SRR072169.449069). Taking into account basal position of Marchantiopsida (particularly, genus *Marchantia*) among land plants [25, 32, 34] (Figure 3), it can be proposed that the ancient miR390-dependent TAS molecular machinery firstly evolved soon after the transition of plants from water to land. Importantly, using *A. thaliana *protein mRNAs that are involved in different ta-siRNA maturation steps as queries for BLAST searches in *Marchantia polymorpha* random whole genome shotgun library at NCBI identified (as it was previously found for *P. patens* [36]) presence of sequences coding for fragments of some proteins involved in small RNA pathways guided by miR390 (data not shown), namely, AtDCL4 (e.g., NCBI accession number SRR072169.369752.2) and AtRDR6 (e.g., SRR072165.261593.2 and SRR072167.366791.2).

#### 3.1.2. Search for Novel TAS3 Genes in Bryophyta and Marchantiophyta

**Figure 4:**
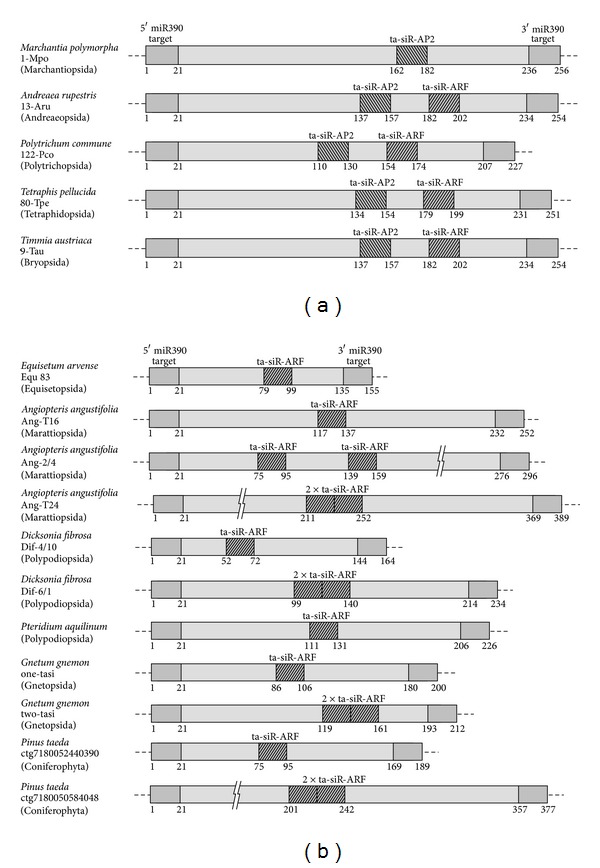
Comparison of TAS3 internal organization between nonflowering plants. Numbers below boxes show relative nucleotide positions of miR390 target sites and ta-siRNAs. (a) Organization of TAS3 loci in Bryophyta and Marchantiophyta. (b) Organization of TAS3 loci in ferns and gymnosperms. For other details see text.

In a previous paper we used PCR amplification with designed degenerated primers BryoTAS-P and BryoTAS-M as a comparative profiling tool to probe genomic DNA samples derived from 13 moss species [24]. Our data on miR390 detection in mosses and liverworts (see above) suggested further search for TAS3 genes in lower land plants. Particularly, we tested moss species from *Timmia austriaca* (class Bryopsida, subclass Timmiidae), *Tetraphis pellucida *(Tetraphidopsida), *Polytrichum commune* (Polytrichopsida), and *Andreaea rupestris* (Andreaeopsida). In all these species one or more potential TAS3 loci were detected (Figures 3 and 4, Table 1). The detection approach was principally identical to that described in our previous paper [24]. In addition, we sequenced a dozen of new TAS3 species in the representatives of class Bryopsida (Table 1). In general, identification of around 40 new TAS3 species in four classes of mosses including the early diverged Andreaeopsida [25, 34] showed that all sequenced TAS3 loci fit general structural organization of *Physcomitrella* genes, namely, dual miR390 target sites on the borders and monomeric tasiARF/tasiAP2 sequences between them (Figure 4(a) and Table 1). Interestingly, recent studies on *Physcomitrella* TAS3 loci showed that tasiAP2 sequences in PpTAS3c and PpTAS3e do not appear functional because of a number of substitutions preventing Watson-Crick pairing with target mRNA (see Table S3 in [20]). This observation allows us to put forward the hypothesis that evolution of TAS3 loci toward the tasiARF only species typical for flowering plants involved selection of those diverged moss TAS3 genes that have lost functional tasiAP2 sequences. 

Our data on miR390 detection in Marchantiophyta (see above) allowed us to propose that miR390-dependent molecular machinery should act in these basal land plants. It is of great importance to reveal the role of miR390 in early land plant evolution. It seems this role is more complicated than simple triggering of the TAS3 precursor processing if we assume potential involvement of miR390 in the direct targeting and cleavage of protein-coding mRNAs [41, 42]. This potential complicated role is seemingly illustrated by our data showing that despite the finding of miR390-like sequence in* Harpanthus flotovianus* and *Oedipodium griffithianum *(Figure 1), we failed to detect TAS3 species in these plants (data not shown). In contrast, PCR-based approach with BryoTAS-P/BryoTAS-M primers and *Marchantia* total genomic DNA revealed a weak but reproducible amplified DNA fragment of 250 bp (data not shown). Cloning and sequencing of this PCR product showed TAS3-like sequence which was also found among genome reads of *Marchantia polymorpha* genome by BLAST search of NCBI SRA database (NCBI accession number SRR072168.997878) (Table 1). Strikingly, the TAS3-like liverwort locus contains only monomeric tasiAP2 site and no tasiARF sequences (Figure 4(a)). Assuming basal position of Marchantiopsida among land plants [25, 32, 34], it can be proposed that the ancient plant miR390-dependent TAS molecular machinery firstly evolved to target AP2-like mRNAs and only then both ARF- and AP2-specific mRNAs. 

**Figure 5:**
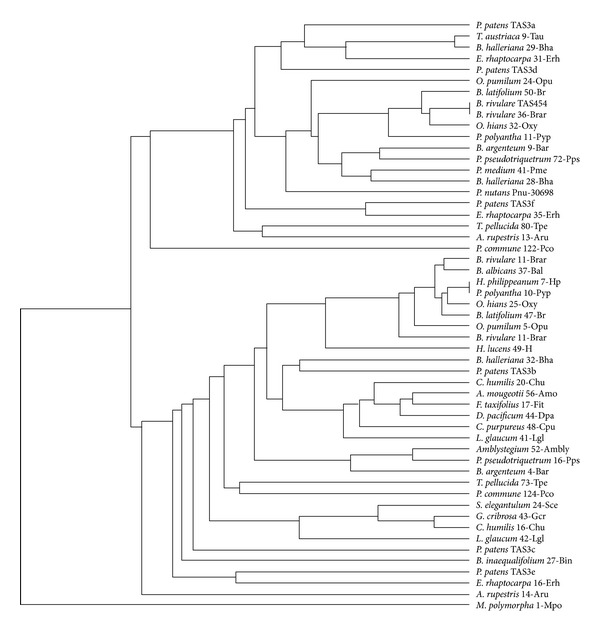
The minimal evolution phylogenetic tree based on analysis of the aligned TAS3 genes from mosses. This tree was generated according MAFFT6 program. For full plant names and accession numbers see Table 1.

Recently, analysis of the *Physcomitrella patens *RNA degradome revealed the novel moss TAS loci, TAS6, which are dependent on the activity of dual sites complementary to miR156/miR529 [20]. All three revealed TAS6 loci are positioned in close genomic proximity to PpTAS3 loci (viz., PpTAS3a, PpTAS3d, and PpTAS3f) (Table 1) and expressed as common RNA precursors with these TAS3 species (Table 1) [15, 20, 38]. Moreover, miR156 influences accumulation of tasiRNAs specific for PpTAS3a [38]. We found that proximity of TAS6 loci to TAS3 genes is not unique for *Physcomitrella patens* (subclass Funariidae). These TAS gene clusters are present also in the mosses of subclasses Bryidae and Dicranidae (Table 1). Importantly, most TAS3 sequences potentially expressed as common precursors with TAS6 species form common cluster on the moss TAS3 phylogenetic dendrogram with PpTAS3a, PpTAS3d, and PpTAS3f (TAS3-like locus of *Marchantia polymorpha* was used as outgroup) (Figure 5 and Table 1). This dendrogram also showed that TAS3 sequences from representatives of Tetraphidopsida, Polytrichopsida, and Andreaeopsida positioned mostly as basal molecular species in two main moss TAS3 clusters (Figure 5). 

#### 3.1.3. TAS3 Genes in Ferns and Gymnosperms

Gymnosperms are different from angiosperms in peculiarities of seed formation and flowering. Recent works on gymnosperm miR390-based gene regulation have focused mainly on discovery and functional analysis of miRNA [6, 9]. Unlike TAS3 of flowering plants, it seems that only the 5′miR390-target site is cleaved in conifer TAS3-like RNA precursor [15, 29]. Our screening of NCBI and other databases for TAS3 sequences showed two TAS3 subfamiles (one-tasiARF and two-tasiARF) in many representatives Coniferophyta (our unpublished data). Particularly, the *Pinus taeda* genome contains at least 4 one-tasiARF TAS3 loci (sequences from clones deg7180055903842, ctg7180052440390, ctg7180045727317, and ctg7180039236567) and 7 two-tasiARF TAS3 loci (sequences from clones ctg7180050584048, ctg7180047037037, ctg7180045493386, >ctg7180064976704, ctg7180063936100, ctg7180044268127, and ctg7180058353378) (http://dendrome.ucdavis.edu/resources/blast/) (Figure 4(b) and data not shown). Likewise, the plants from Gnetophyta (viz., *Welwitschia mirabilis *and *Gnetum gnemon*) code for TAS3 loci belonging to one-tasiARF (NCBI accession number CK744773 and SRR064399.239159) and two-tasiARF (SRR064399.294117 and SRR064399.72564) (Figure 4(b)). To further reveal the conservation of TAS3-like loci in lower seed plants, we performed PCR amplification of total DNA from Cycadophyta (*Cycas revoluta*, *Strangeria eriupus*, *Zamia pumila*, and *Macrozamia miqnolii*) using dicot-specific primers P-Tas3, M-Tas3/caa, and M-Tas3/aca [23]. Sequencing and BLAST analysis of the obtained PCR products revealed that they corresponded to the monomeric and dimeric ta-siARF precursors of flowering plants (data not shown). Thus, the available data on sequencing the TAS3 genes from gymnosperms unambiguously showed two TAS3 subfamiles related to those found in flowering plants (Figure 4(b)). 

In general, first tracheophytes are proposed to have evolved >420 million years ago, and a major innovation in the evolution of tracheophytes from their bryophyte-like ancestors was the ability to form supporting and conducting tissues that contain cells with lignified cell walls [32, 34]. The ferns comprise one of the most ancient tracheophytic plant lineages and occupy habitats ranging from tundra to deserts and the equatorial tropics. Like their nearest relatives (viz., conifers) extant ferns possess tracheid-based xylem. Little is known about the miR390/TAS3-based formation of tasiRNAs in ferns [43], and the selection pressures that influenced the structure of TAS3 genes in diverse taxonomic groups of ferns and fern allies (particularly, classes Lycopodiopsida, Equisetopsida, Marattiopsida, and Polypodiopsida) remain obscure. For example, it was unexpectedly found that the genome of *Selaginella moellendorffii* from Lycopodiopsida lacks DCL4, RDR6, TAS3, and MIR390 loci, which are required for the biogenesis of ta-siARF RNAs [44]. 

**Figure 6:**
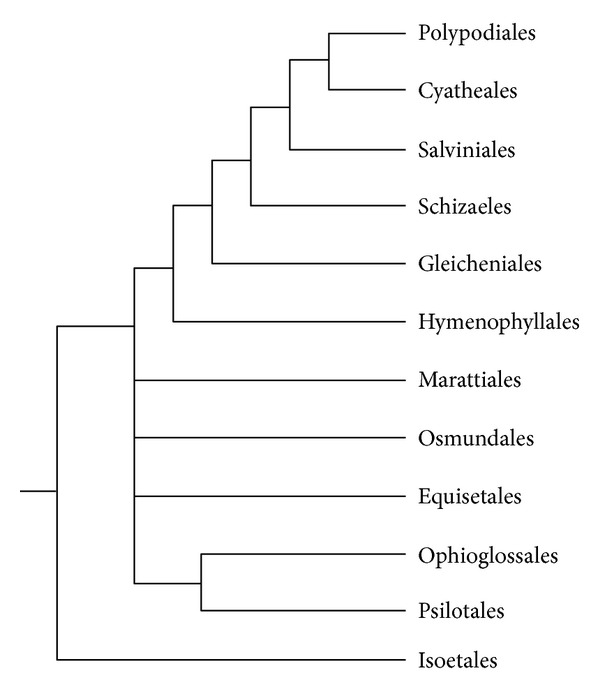
Synoptic consensus view on phylogeny of major fern taxa based on recent molecular analyses, adopted from Figure 2 of Sen et al. [26] with modifications. Note that Isoetales species are outside ferns.

To achieve an understanding on the evolutionary history of TAS3 genes in ferns, we focused on two aims (1) identifying possible TAS3 genes in 4 fern orders and (2) unraveling the pattern of TAS3 sequence organization among fern lineages. We choose two earliest diverged orders (Marattiales and Equisetales), which positioned relatively close to the common ancestor of ferns and seed plants, as well as two core leptosporangiates orders (Polypodiales and Cyatheales) (Figure 6) [26, 45]. Using dicot-specific primers P-Tas3, M-Tas3/caa, and M-Tas3/aca (see above) allowed us to find that plants of class Polypodiopsida (Polypodiales) encode both one- and two-tasiARF TAS3 subfamilies like gymnosperms and angiosperms (Figure 4(b)). However, sequencing of amplified TAS3 loci from Equisetopsida (Equisetales) revealed only one-tasiARF TAS3 species (Figure 4(b)). *Angiopteris angustifolia* (Marattiopsida) showed single two-tasiARF TAS3 locus, which included the tandem of conserved tasiARF sequences corresponding to the nearby phased segments defined by the 3′ miR390 processing site, as observed in *Arabidopsis* and some other plant species [14, 27]. Strikingly, another peculiar two-tasi locus also encoded two tasiARF's which are separated by the spacer sequence (Figure 4(b)). It is tempting to speculate that two-tasiARF subfamily possessing spacer between tasiARF sequences may represent an intermediate evolutionary stage from one-tasiARF to tandem tasiARF organization of TAS3 loci.

#### 3.1.4. Peculiar Evolution of TAS3 Genes in Land Plants

Similarity of miR390-triggered siRNA regulation of angiosperm and bryophyte ARF transcripts could be regarded as evidence that all modern TAS3 genes might have descended from a common ancestor [46]. Importantly, it was recently revealed that all TAS3 genes may have important and complex roles in the evolution of plant developmental processes and biotic stress response [5, 15, 33, 38]. However, a vast majority of higher plant ARF3 and ARF4 mRNAs possessed dual complementary sites to TAS3-derived ta-siRNAs, whereas the *P. patens* ARF mRNA possessed only one site complementary to TAS3 ta-siRNAs. As it was stated by Axtell et al. [46] “These observations add to the examples of convergent evolutionary origins for similarly functioning small RNAs, while illustrating that cleavage of a homologous target at an analogous location does not always indicate descent from a common ancestor. Moreover, the ARF-targeting ta-siRNAs are derived from the (+) strand of the angiosperm TAS3 loci, whereas they derived from the (–) strands of the moss TAS3 loci, suggesting a more complex evolutionary path for plant TAS3 loci than simple sequence divergence from a common ancestor. Data from additional plant lineages might provide important clues for distinguishing between these interesting possibilities.”

Our data on additional plant lineages indicate that the main mechanism of TAS3 gene evolution that could generate diversity is the duplication and divergence of TAS3 loci. After appearance of the putative primitive (AP2-specific) TAS3-like locus, this growing gene family must have undergone copy number gains upon evolutionary flow toward mosses, and one of the duplicates may be subjected to neofunctionalization resulted in evolving tasiARF sequences in addition to tasiAP2 site (Figure 4(a), Table 2). Our analysis suggests that moss TAS3 families may undergone losses of tasiAP2 sites during evolution toward ferns. As a result, horsetails seem to preserve only one-tasiARF TAS3 loci. Importantly, unlike mosses ARF-targeting ta-siRNAs are derived from the (+) strand of the fern TAS3 precursor RNA, suggesting complex evolutionary events toward tracheophyte TAS3 species [46] resulted, particularly, in complete deleting miR390-based machinery from lycophyte genomes [44]. Finally, the evolutionary process to build up angiosperm-type TAS3 sequences may be related to step-by-step duplications of monomeric tasiARF sites first observed in *Angiopteris angustifolia* (Figure 4, Table 2). Interestingly, “core consensus” in one-tasiARF species was found to be different from consensus in two-tasi TAS3 [23]. Although the significance of this variation remains obscure, we observed the same phenomenon in fern TAS3 species (data not shown). 

### 3.2. Differential Expression of TAS3-Like Genes from the Tribe Senecioneae

Global transcriptome nextgeneration sequencing studies in many plant species have shown shifts in the pools of the different ta-siRNAs and miRNA classes in various tissues and organs [47] (http://smallrna.udel.edu/). In general, plant cells have a small number of unique and highly abundant 21 nt miRNAs/ta-siRNAs and a large number of diverse siRNAs, mainly 24 nt in size [1]. A comprehensive analysis can be compiled on variation in the miRNAs present in a large population of dozens of millions sequences derived from several libraries representing multiple tissues/organs of the plant [48]. Around several thousand unique signatures corresponding to miRNAs were detailed for some plants. Examination of the number of reads of each signature may reveal all the length or sequence variations found in the high throughput populations for all miRNA family members. Thus, the flux of expression patterns for each of the unique signatures in the libraries representing both seed and vegetative tissues are currently detailed in many species. Some of the miRNAs were very abundant in certain tissues such as members of the miR167 family, which were highly prevalent in the seed coats from multiple samples. Some other miRNAs showed preferential expression in either of the flowers, germinating cotyledons, stems, or leaves. In summary, it is clear that tissue differences in normalized read counts are apparent in many of the known miRNAs and ta-siRNAs that are conserved widely in plant systems [1, 48, 49].

**Figure 7:**
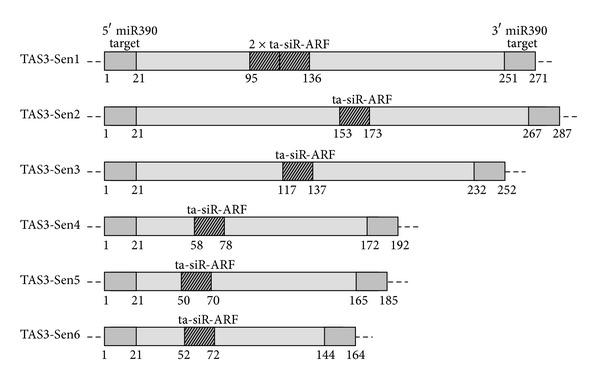
Comparison of TAS3 internal organization in plants belonging to subtribe Senecioneae. Numbers below boxes show relative nucleotide positions of miR390 target sites and ta-siRNAs. For other details see text.

Senecioneae is the largest tribe of family *Asteraceae*, comprised of ca. 150 genera and 3,000 species which include many common succulents of greenhouses. Approximately one-third of species are placed in genus *Senecio*, making it one of the largest genera of flowering plants [50]. However, there was no information on the structure of TAS genes in these plants. Recently, we revealed that the conserved TAS3 locus (TAS3-Sen1) in *Senecio talinoides*, *Curio repens*, and *Curio articulatus* was actively transcribed, and its close relatives are distributed among many *Asteraceae* plants and found to be similar to Arabidopsis AtTAS3a gene [51]. To reveal the possible novel TAS3-like loci in these plants, we performed PCR amplification of total DNA using dicot-specific primers P-Tas3, M-Tas3/caa, and M-Tas3/aca. As it was found for tobacco [23], PCR on Senecioneae DNA gave rise to one major DNA fragment of 250 bp and, in addition, smaller minor bands including those of 170–190 bp in length (data not shown). Sequencing of these PCR DNA fragments revealed that they corresponded to five new one-tasiARF and two-tasiARF TAS3 precursors (Figure 7). BLAST analysis of NCBI databases revealed their distant relation to the sequenced TAS3-Sen1 ta-siARF precursors from *Asteracea*, (data not shown).

We have studied the transcription of TAS3-Sen1 genes in three above mentioned Senecioneae species by sequence analysis of multiple cDNA clones from vegetative organs (roots, true leaves, and succulent leaves). Since reverse transcription priming with the oligo (dT) at the poly A tails of the TAS3 genes analyzed may not occur with the same efficiency, we used correct priming with six-specific primers during PCR on the total cDNA preparation. Thus, the cDNA clones obtained should represent true qualitative measure of the specific TAS3 RNAs. Unexpectedly, we found eight cDNA clones for *C. repens *TAS3-Sen1, seven clones for *C. articulatus* TAS3-Sen1, and five clones for *Senecio talinoides* TAS3-Sen1 but no TAS3 clones for five other TAS3 species (TAS3-Sen2 to TAS3-Sen6) (data not shown). In accordance with our experimental data bioinformatic analysis of SRA database of *Senecio madagascariensis* transcriptome at NCBI revealed only TAS3-Sen1 read (GenBank accession number SRR006592) (data not shown). Thus, despite the presence of at least six TAS3 genes in representatives of subtribe Senecioneae, a single locus is transcribed in the vegetative organs. 

## Figures and Tables

**Table 1:** Description of sequenced TAS3-like loci in mosses.

Class/subclass	Order	Species	^ a^Clone/cluster	^ b^Length, bp	^ c^Reference and/or accession number	^ d^Organization (one- or two-tasiARF/AP2/TAS6)
Bryopsida/Dicranidae	Pottiales	*Bryoerythrophyllum inaequalifolium *	27-Bin/ *TAS3b,c,e *	236	KC812740	tasiARF/AP2
	Gremiales	*Coscinodon humilis *	20-Chu/ *TAS3b,c,e *	234	Krasnikova et al., 2011 [24] HQ709423	—“—
		*C. humilis *	16-Chu/ *TAS3b,c,e *	228	Krasnikova et al., 2011 [24] HQ709422	—“—
		*Schistidium elegantulum *	24-Sce/ *TAS3b,c,e *	229	Krasnikova et al., 2011 [24] HQ709424	—“—
		*Grimmia cribrosa *	43-Gcr/ *TAS3b,c,e *	228	Krasnikova et al., 2011 [24] HQ709421	—“—
	Dicranales	*Fissidens taxifolius *	17-Fit/ *TAS3b,c,e *	192	Krasnikova et al., 2011 [24] HQ709425	—“—
		*Ceratodon purpureus *	48-Cpu/ *TAS3b,c,e *	231 779^e^	SRR074890.1165977.2	tasiARF/AP2/ TAS6
		*Amphidium mougeotii *	56-Amo/ *TAS3b,c,e *	234	KC812745	tasiARF/AP2
		*Leucobryum glaucum *	41-Lgl/ *TAS3b,c,e *	226	KC812750	—“—
		*L. glaucum *	42-Lgl/ *TAS3b,c,e *	192	KC812749	—“—
		*Dicranum pacificum *	44-Dpa/ *TAS3b,c,e *	232	KC812741	—“—

Bryopsida/Bryidae	Orthotrichales	*Orthotrichum pumilum *	5-Opu/ *TAS3b,c,e *	199	Krasnikova et al., 2011 [24] HQ709419	—“—
		*O. pumilum *	24-Opu/ *TAS3a,d,f *	246	Krasnikova et al., 2011 [24] HQ709420	—“—
	Hypnales	*Brachythecium latifolium *	47-Br/ *TAS3b,c,e *	199	Krasnikova et al., 2011 [24] FJ804748	—“—
		*B. latifolium *	50-Br/ *TAS3a,d,f *	255	Krasnikova et al., 2011 [24] FJ804747	—“—
		*B. albicans *	37-Bal/ *TAS3b,c,e *	199	Krasnikova et al., 2011 [24] HQ709414	—“—
		*B. rivulare *	11-Brar/ *TAS3b,c,e *	199	KC812739	—“—
		*B. rivulare *	36-Brar/ *TAS3a,d,f *	255 1444^e^	KC812738 (Illumina sequence)	tasiARF/AP2/ TAS6
		*Homalothecium philippeanum *	7-Hp/ *TAS3b,c,e *	199	Krasnikova et al., 2011 [24] HQ709415	tasiARF/AP2
		*Amblystegium sp *	52-Ambly/ *TAS3b,c,e *	191	KC812762	—“—
		*Oxyrrhynchium hians *	25-Oxy/ *TAS3b,c,e *	199	Krasnikova et al., 2011 [24] HQ709418	—“—
		*O. hians *	32-Oxy/ *TAS3a,d,f *	253	KC812761	—“—
		*Pylaisia polyantha *	10-Pyp/ *TAS3b,c,e *	199	Krasnikova et al., 2011 [24] HQ709416	—“—
		*P. polyantha *	11-Pyp/ *TAS3a,d,f *	253	Krasnikova et al., 2011 [24] HQ709417	—“—
	Hookeriales	*Hookeria lucens *	49-H/ *TAS3b,c,e *	190	Krasnikova et al., 2011 [24] FJ804749	—“—
	Bryales	*Pohlia nutans *	Pnu-30698 *TAS3a,d,f *	259 920^e^	GACA01023180.1	tasiARF/AP2/ TAS6
		*Plagiomnium medium *	41-Pme/ *TAS3a,d,f *	236	KC812756	tasiARF/AP2
		*Ptychostomum pseudotriquetrum *	72-Pps/ *TAS3a,d,f *	246	KC812758	—“—
		*P. pseudotriquetrum *	16-Pps/ *TAS3b,c,e *	193	KC812757	—“—
		*Bryum argenteum *	9-Bar/ *TAS3a,d,f *	233	KC812760	—“—
		*B. argenteum *	4-Bar/ *TAS3b,c,e *	192	KC812759	—“—
	Bartramiales	*Bartramia halleriana *	32-Bha/ *TAS3b,c,e *	228	KC812748	—“—
		*B. halleriana *	28-Bha/ *TAS3a,d,f *	272	KC812747	—“—
		*B. halleriana *	29-Bha/ *TAS3a,d,f *	254	KC812746	—“—

Bryopsida/Funariidae	Encalyptales	*Encalypta rhaptocarpa *	35-Erh/ *TAS3a,d,f *	253	KC791767	—“—
		*E. rhaptocarpa *	16-Erh/ *TAS3b,c,e *	249	KC791768	—“—
		*E. rhaptocarpa *	31-Erh/ *TAS3a,d,f *	253	KC791769	—“—
	Funariales	*Physcomitrella patens *	PpTAS3a/ *TAS3a,d,f *	255 895^e^	Arif et al., 2012 [20]	tasiARF/AP2/ TAS6
		*P. patens *	PpTAS3d/ *TAS3a,d,f *	256 1250^e^	Arif et al., 2012 [20]	—“—
		*P. patens *	PpTAS3f/ *TAS3a,d,f *	245 1234^e^	Arif et al., 2012 [20]	—“—
		*P. patens *	PpTAS3c/ *TAS3b,c,e *	259	Arif et al., 2012 [20]	tasiARF/AP2
		*P. patens *	PpTAS3b/ *TAS3b,c,e *	192	Arif et al., 2012 [20]	—“—
		*P. patens *	PpTAS3e/ *TAS3b,c,e *	192	Arif et al., 2012 [20]	—“—

Bryopsida/Timmiidae	Timmiales	*Timmia austriaca *	9-Tau/ *TAS3a,d,f *	254	KC812755	—“—

Tetraphidopsida	Tetraphidales	*Tetraphis pellucida *	73-Tpe/ *TAS3b,c,e *	275	KC812754	—“—
		*T. pellucida *	80-Tpe/ outside	251	KC812753	—“—

Polytrichopsida	Polytrichales	*Polytrichum commune *	122-Pco/ outside	227	KC812751	—“—
		*P. commune *	124-Pco/ *TAS3b,c,e *	262	KC812752	—“—

Andreaeopsida	Andreaeales	*Andreaea rupestris *	13-Aru/ outside	254	KC812744	—“—
		*A. rupestris *	14-Aru/ outside	272	KC812743	—“—

Sphagnopsida	Sphagnales	*Sphagnum squarrosum *	Not found	—	Krasnikova et al., 2011 [24]	—
		*S. girgensohnii *	Not found	—	Krasnikova et al., 2011 [24]	—

Marchantiopsida	Marchantiales	*Marchantia polymorpha *	1-Mpo/ outside	256	KC812742 SRR072168.997878.2	tasiAP2

^a^This column includes the names of all identified TAS3 clones (upper line) and clusterization with *Physcomitrella patens* loci on the dendrogram (lower line).

^b^This column indicates the length of all TAS3 species including both 5′ and 3′ miR390 target sequences on the borders.

^c^This column includes the references and accession numbers (if known) for all identified TAS3 loci except *Physcomitrella patens* loci. For loci 48-Cpu and 1-Mpo, our sequence data are identical to the fragments of longer sequences found in NCBI SRA database. For 36-Brar, PCR-based sequence corresponds to the fragments of the longer Illumina reads of *B. rivulare *genomic DNA (Goryunov et al. unpublished).

^d^This column indicates internal organization of TAS3 loci which include tasiARF and tasiAP2 sequences. TAS6 means the occurrence of the particular TAS3 locus in close proximity to TAS6 locus (if known).

^e^This number indicates total length of TAS6-TAS3 complex element (from the 5′ miR529 target site in TAS6 to 3′ miR390 target site in TAS3).

**Table 2:** Summary of the diversity in the organization of TAS3-like loci.

Organization of TAS3 (One- or two-tasiARF/AP2/TAS6)	Class/subclass
One-tasiAP2 only	Marchantiopsida
One-tasiAP2-one-tasiARF	Andreaeopsida Polytrichopsida Tetraphidopsida Bryopsida/Timmiidae Bryopsida/Funariidae Bryopsida/Bryidae Bryopsida/Dicranidae
TAS6-TAS3	Bryopsida/Funariidae Bryopsida/Bryidae Bryopsida/Dicranidae
One-tasiARF only	Equisetopsida
One-tasiARF and two-tasiARF species	Marattiopsida
Two-tasiARF (no tandem repeat)	Marattiopsida
One-tasiARF and two-tasiARF species	Polypodiopsida
One-tasiARF and two-tasiARF species	Spermatophyta*
No TAS3 loci revealed	Lycopodiopsida Sphagnopsida

See text for details. *Spermatophyta species form a group, which includes several classes.
